# Cesarean Delivery Trends Among Patients at Low Risk for Cesarean Delivery in the US, 2000-2019

**DOI:** 10.1001/jamanetworkopen.2023.5428

**Published:** 2023-03-29

**Authors:** Anna M. Frappaolo, Teresa C. Logue, Dena Goffman, Lisa M. Nathan, Jean-Ju Sheen, Maria Andrikopoulou, Timothy Wen, Mary E. D’Alton, Alexander M. Friedman

**Affiliations:** 1 Division of Maternal-Fetal Medicine, Department of Obstetrics and Gynecology, Columbia University College of Physicians and Surgeons, New York, New York; 2 Department of Obstetrics and Gynecology, Christiana Care Health System, Newark, Delaware; 3 Department of Obstetrics, Gynecology, and Reproductive Sciences, University of California, San Francisco

## Abstract

**Importance:**

Reducing rates of unnecessary cesarean deliveries is both a national and a global health objective. However, there are limited national US data on trends in indications for low-risk cesarean delivery.

**Objective:**

To determine temporal trends in and indications for cesarean delivery among patients at low risk for the procedure over a 20-year period.

**Design, Setting, and Participants:**

This cross-sectional study analyzed 2000 to 2019 delivery hospitalizations using the National Inpatient Sample. Births at low risk for cesarean delivery were identified using a definition from the Society for Maternal-Fetal Medicine and additional criteria. Temporal trends in cesarean birth were analyzed using joinpoint regression to estimate the average annual percentage change (AAPC) with 95% CIs. Data analysis was performed from August 2022 to January 2023.

**Exposure:**

This analysis evaluated cesarean birth trends in a population at low risk for this procedure over a 20-year period.

**Main Outcomes and Measures:**

In addition to overall cesarean birth risk, cesarean deliveries for nonreassuring fetal status and labor arrest were individually analyzed.

**Results:**

Of an estimated 76.7 million delivery hospitalizations, 21.5 million were excluded according to the Society for Maternal-Fetal Medicine definition, and 14.7 million were excluded according to additional criteria. Of the estimated 40 517 867 deliveries included, 12.1% (4 885 716 deliveries) were by cesarean delivery. Cesarean deliveries among patients at low risk for the procedure increased from 9.7% to 13.9% between 2000 and 2009, plateaued, and then decreased from 13.0% to 11.1% between 2012 and 2019. The AAPC for cesarean delivery was 6.4% (95% CI, 5.2% to 7.6%) from 2000 to 2005, 1.2% from 2005 to 2009 (95% CI, −1.2% to 3.7%), and −2.2% from 2009 to 2019 (95% CI, −2.7% to −1.8%). Cesarean delivery for nonreassuring fetal status increased from 3.4% of all deliveries in 2000 to 5.1% in 2019 (AAPC, 2.1%; 95% CI, 1.7% to 2.5%). Cesarean delivery for labor arrest increased from 3.6% in 2000 to a peak of 4.8% in 2009 before decreasing to 2.7% in 2019. Cesarean deliveries for labor arrest increased during the first half of the study (2000-2009) for the active phase (from 1.5% to 2.1%), latent phase (from 1.1% to 1.5%), and second stage (from 0.9% to 1.3%) and then decreased from 2010 to 2019, from 2.1% to 1.7% for the active phase, from 1.5% to 1.2% for the latent phase, and from 1.2% to 0.9% for the second stage.

**Conclusions and Relevance:**

Cesarean deliveries among patients at low risk for cesarean birth appeared to decrease over the latter years of the study period, with cesarean deliveries for labor arrest becoming less common.

## Key Points

**Question:**

What are trends in and indications for cesarean delivery among patients at low risk for this procedure in the US?

**Findings:**

This cross-sectional study including more than 40 million deliveries found that cesarean deliveries among patients at low risk for the procedure increased from 9.7% to 13.9% between 2000 and 2009, plateaued, and then decreased from 13.0% to 11.1% between 2012 and 2019. Cesarean delivery for nonreassuring fetal status increased over the study period, whereas cesarean delivery for labor disorders decreased over the latter half of the study period.

**Meaning:**

These findings suggest that there are countervailing trends in cesarean delivery, with diagnoses for labor disorders decreasing and diagnoses for nonreassuring fetal status increasing.

## Teaser

This cross-sectional study examines temporal trends in and indications for cesarean births among patients at low risk for this procedure in the US from 2000 to 2019.

## Introduction

When clinically indicated, cesarean delivery may improve both maternal and neonatal outcomes. However, when not clinically indicated, cesarean delivery is a major surgical intervention that increases risk for adverse outcomes.^1^ Reducing unnecessary cesarean delivery rates is both a national and a global health objective. The US Department of Health’s Healthy People 2030 campaign has identified the reduction of cesarean births as a primary objective, with the goal of reducing the rate of cesarean births among low-risk, nulliparous patients to 23.6% by the year 2030.^2^ In 2012, a joint workshop including the National Institute of Child Health and Human Development identified clinically modifiable factors that could decrease cesarean delivery rates, including diagnosing labor arrest only after adequate time had elapsed.^3^ The American College of Obstetricians and Gynecologists and Society for Maternal-Fetal Medicine (SMFM) subsequently made specific recommendations for diagnosing labor arrest.^1^ Since this guidance, several studies^4,5,6,7,8^ have examined trends in cesarean delivery rates and maternal and neonatal outcomes. An analysis^9^ of births in California found that nulliparous, full-term, singleton, vertex cesarean births decreased from 2014 to 2019, in the context of a statewide quality improvement effort. However, there are limited national data on trends in indications for cesarean delivery. The national outcomes of changes in clinical practice, particularly with respect to diagnosis of labor arrest, are unknown. Given these knowledge gaps, the purposes of this study were to (1) determine temporal trends in cesarean deliveries among patients at low risk for cesarean delivery over a 20-year period in the US and (2) analyze trends in specific cesarean delivery indications.

## Methods

### Study Design and Sample

This repeated cross-sectional analysis was restricted to delivery hospitalizations for patients aged 15 to 39 years who were at low risk for cesarean delivery. Delivery hospitalizations were identified using algorithms of *International Classification of Diseases, Ninth Revision, Clinical Modification (ICD-9-CM*) and *International Statistical Classification of Diseases, Tenth Revision, Clinical Modification (ICD-10-CM)* codes that have previously been shown to capture more than 95% of deliveries.^10,11^ Delivery hospitalizations at low risk for cesarean delivery were identified using *ICD-9-CM* and *ICD-10-CM *criteria from the SMFM.^12,13^ The SMFM criteria define deliveries as low risk for cesarean delivery if they are full term with singleton, vertex fetuses, there is no history of prior cesarean delivery, there are no absolute or relative contraindications to vaginal birth, and there are no other conditions associated with increased risk for cesarean delivery, such as HIV infection, placenta previa, prior cesarean delivery, eclampsia, congenital anomalies, HELPP (hemolysis, elevated liver enzymes, and low platelets) syndrome, and maternal adult congenital cardiovascular disease, among other diagnoses. We further excluded delivery hospitalizations associated with diagnoses of obesity, chronic hypertension, pregestational and gestational diabetes, preeclampsia, and postdates gestational age. We excluded these additional conditions because they are associated with an increased risk for cesarean delivery, are common on a population basis, may be affected by temporal trends, and may contribute to the overall cesarean delivery risk estimates in a population.^14,15^

### Data Collection

We identified delivery hospitalizations of patients at low risk for cesarean delivery within the US National Inpatient Sample (NIS) created by the Agency for Healthcare Research and Quality’s Healthcare Cost and Utilization Project from 2000 through 2019. The NIS comprises approximately 20% of all hospitalizations nationally and is one of the largest publicly available, all-payer inpatient databases in the US.^16^ From 2000 to 2011, the NIS included all hospitalizations from individual hospitals and, from 2012 on, used a systematic sampling design with proportionate discharges from each hospital.^17,18,19^ Population weights provided by the Healthcare Cost and Utilization Project can be applied to the NIS to create national estimates for trends; these weights were applied for this study.^20^ On October 1, 2015, NIS coding transitioned from *ICD-9-CM* to *ICD-10-CM* coding. To perform analyses across the study period while accounting for this coding change, *ICD-9-CM* codes were translated to *ICD-10-CM* codes using the publicly available General Equivalence Mappings provided by the Centers for Medicare & Medicaid Services and the National Center for Health Statistics.^21^

Given that this study involved a deidentified and publicly available data set, the Columbia University institutional review board deemed this study exempt from review and the need for informed consent (the Columbia institutional review board determination was Research of Existing Data/Records/Specimens–46.101(b)4). We followed the Strengthening the Reporting of Observational Studies in Epidemiology (STROBE) reporting guidelines for cross-sectional studies for this analysis.^22^

### Exposures

Demographic factors included year of delivery, maternal age, self-reported maternal race and ethnicity, payer, and median income quartile based on ZIP code. Race and ethnicity were assessed in this study because of the potential for disparities related to this exposure. Hospital characteristics included location and teaching status (urban teaching, urban nonteaching, and rural) and geographic region (Northeast, Midwest, South, or West).

### Outcomes

This study had 2 primary objectives. The first objective was to determine temporal trends in cesarean deliveries among all deliveries at low risk for cesarean birth. For the second objective, we sought to determine trends in cesarean delivery specifically for the following diagnoses: (1) nonreassuring fetal status, (2) labor arrest, and (3) obstructed labor (including disproportion). These categories were organized hierarchically and were mutually exclusive such that nonreassuring fetal status included patients with additional diagnoses of labor arrest and obstructed labor. Similarly, labor arrest included diagnoses of obstructed labor (eTable 1 in Supplement 1). In addition to analyzing overall labor arrest trends, we analyzed trends separately for arrest during the latent phase of labor, the active phase of labor, and the second stage of labor. Arrest was categorized hierarchically as second stage arrest, active phase arrest, and latent phase arrest.

### Statistical Analysis

 Data analysis was performed from August 2022 to January 2023. The proportion of low-risk deliveries occurring by cesarean delivery each year was identified, and trends analysis during the study period (2000-2019) was conducted using the National Cancer Institute’s Joinpoint Regression Program (version 4.8.0.1). This program uses linear segmented regression and logarithmic transformation to determine the average annual percentage change (AAPC) with 95% CIs.^23,24^ This program allows identification of when a trend change is produced and calculates the annual percentage change in rates between trend-change points. The program also estimates the AAPC over the whole study period.^25,26^ Joinpoint regression analysis was also performed for the aforementioned cesarean delivery indications.

As an ancillary analysis, the association between demographics, hospital characteristics, and risk for cesarean delivery among patients at low risk for cesarean deliveries was determined. Unadjusted odds for cesarean birth were estimated using logistic regression models accounting for survey design. Adjusted models accounting for survey design were then performed accounting for the previously mentioned demographic and hospital covariates. We calculated unadjusted odds ratios (ORs) and adjusted ORs (aORs) with 95% CIs as measures of association. Two-tailed *P* < .05 was considered significant.

Demographic and hospital factors and the likelihood of cesarean delivery were compared between groups by analyzing the absolute standardized mean difference. A value greater than 0.1 (10%) was interpreted as a meaningful magnitude of difference between the 2 groups.^27^ All analyses were performed with SAS statistical software version 9.4 (SAS Institute), with the exception of the temporal trends analysis performed with the Joinpoint Regression Program.

## Results

From 2000 to 2019, an estimated 76.7 million delivery hospitalizations were identified. Of these, 21.5 million were excluded on the basis of SMFM criteria, and an additional 14.7 million were excluded on the basis of the additional diagnoses of obesity, diabetes, chronic hypertension, gestational hypertension, preeclampsia, and postdates gestation. After applying exclusion criteria, an estimated 40 517 867 deliveries at low risk for cesarean delivery were included in the analysis, of which 12.1% (4 885 716 deliveries) were cesarean deliveries. Cesarean deliveries were more common among older patients (10.0% of vaginal deliveries [3 578 618 deliveries] vs 12.3% of cesarean deliveries [600 881 deliveries] were to patients aged 35 to 39 years), patients with commercial insurance (51.1% of vaginal deliveries [18 208 771 deliveries] vs 55.6% of cesarean deliveries [2 718 592 deliveries] were to patients with commercial insurance), and hospitals in the South (37.7% of vaginal deliveries [13 437 388 deliveries] vs 44.1% of cesarean deliveries [2 153 255 deliveries] were to patients delivering at hospitals in the South) (Table 1).

**Table 1.** Characteristics of Study Population by Mode of Delivery

Characteristic	Participants, No. (%) [95% CI]		SMD^a^
	Vaginal delivery	Cesarean delivery	
Maternal race and ethnicity			
Hispanic	6 749 417 (18.9) [18.0-19.9]	861 368 (17.6) [16.6-18.6]	0.12
Non-Hispanic Black	3 726 905 (10.5) [10.0-10.9]	615 598 (12.6) [12.1-13.1]	
Non-Hispanic White	15 756 548 (44.2) [43.1-45.3]	2 184 261 (44.7) [43.6-45.8]	
Other^b^	3 143 414 (8.8) [8.4-9.3]	451 292 (9.2) [8.8-9.7]	
Unknown	6 255 867 (17.6) [16.2-18.9]	773 197 (15.8) [14.5-17.1]	
Age category, y			
15-17	3 768 916 (10.5) [10.4-10.8]	544 447 (11.1) [10.9-11.4]	0.07
18-24	9 435 620 (26.5) [26.2-26.8]	1 219 604 (25.0) [24.6-25.3]	
25-29	10 419 132 (29.2) [29.1-29.4]	1 350 432 (27.6) [27.5-27.8]	
30-34	8 429 866 (23.7) [23.3-24.0]	1 170 352 (24.0) [23.6-24.3]	
35-39	3 578 618 (10.0) [9.8-10.3]	600 881 (12.3) [12.0-12.6]	
Payer status			
Medicare	169 695 (0.5) [0.4-0.5]	28 672 (0.6) [0.5-0.6]	0.21
Medicaid	14 983 529 (42.1) [41.2-42.9]	1 868 215 (38.2) [37.3-39.2]	
Commercial insurance	18 208 771 (51.1) [50.1-52.1]	2 718 592 (55.6) [54.6-56.7]	
Self-pay	1 168 589 (3.3) [3.0-3.6]	124 870 (2.6) [2.4-2.7]	
Other	1 101 568 (3.1) [2.9-3.3]	145 366 (3.0) [2.8-3.2]	
Median income quartile by ZIP code			
1 (Lowest)	8 359 366 (23.5) [22.7-24.3]	1 182 319 (24.2) [23.2-25.2]	0.09
2	8 760 736 (24.6) [24.0-25.2]	1 160 103 (23.7) [23.1-24.4]	
3	8 731 850 (24.5) [24.0-25.0]	1 184 515 (24.2) [23.7-24.8]	
4 (Highest)	9 243 153 (25.9) [24.8-27.1]	1 280 708 (26.2) [24.8-27.6]	
Missing	537 045 (1.5) [1.3-1.7]	78 072 (1.6) [23.2-25.2]	
Hospital location			
Rural	4 382 219 (12.3) [11.7-12.9]	582 350 (11.9) [11.2-12.6]	0.15
Urban			
Nonteaching	13 894 267 (39.0) [37.6-40.4]	1 981 987 (40.6) [38.9-42.2]	
Teaching	17 248 715 (48.4) [46.9-49.9]	2 307 546 (47.2) [45.5-48.9]	
Missing	106 950 (0.3) [0.2-0.4]	13 833 (0.3) [0.1-0.42]	
Hospital region			
Northeast	5 414 818 (15.2) [14.3-16.1]	797 849 (16.3) [15.3-17.4]	0.15
Midwest	7 920 003 (22.2) [21.2-23.3]	913 188 (18.7) [17.7-19.7]	
South	13 437 388 (37.7) [36.2-39.2]	2 153 255 (44.1) [42.3-45.8]	
West	8 859 942 (24.9) [23.6-26.2]	1 021 424 (20.91) [19.6-22.2]	
Year			
2000	232 6615 (6.5) [5.9-7.2]	248 484 (5.1) [4.6-5.6]	0.28
2001	2 057 836 (5.8) [5.2-6.3]	226 448 (4.6) [4.2-5.1]	
2002	2 058 110 (5.8) [5.2-6.3]	244 130 (5.0) [4.5-5.5]	
2003	1 992 147 (5.6) [5.1-6.1]	267 022 (5.5) [4.9-6.0]	
2004	2 002 482 (5.6) [5.1-6.1]	279 181 (5.7) [5.1-6.3]	
2005	1 971 545 (5.5) [5.0-6.1]	291 229 (6.0) [5.3-6.6]	
2006	1 973 104 (5.5) [5.0-6.1]	295 473 (6.1) [5.4-6.6]	
2007	2 020 840 (5.7) [5.1-6.2]	313 784 (6.4) [5.7-7.1]	
2008	1 862 438 (5.2) [4.7-5.8]	290 479 (6.0) [5.9-0.3]	
2009	1 796 659 (5.0) [4.5-5.6]	290 399 (5.9) [5.9-0.3]	
2010	1 675 704 (4.7) [4.2-5.2]	250 135 (5.1) [5.1-0.3]	
2011	1 637 656 (4.6) [4.1-5.1]	243 732 (5.0) [5.0-0.3]	
2012	1 654 160 (4.6) [4.4-4.9]	246 730 (5.1) [4.7-5.4]	
2013	1 622 359 (4.6) [4.3-4.8]	232 130 (4.8) [4.4-5.1]	
2014	1 617 995 (4.5) [4.3-4.8]	220 750 (4.5) [4.2-4.8]	
2015	1 562 660 (4.4) [4.1-4.6]	206 715 (4.2) [4.0-4.5]	
2016	1 512 028 (4.2) [4.0-4.5]	196 905 (4.0) [3.8-4.3]	
2017	1 453 863 (4.1) [3.8-4.3]	186 395 (3.8) [3.6-4.1]	
2018	1 412 774 (4.0) [3.7-4.2]	177 765 (3.6) [3.4-3.9]	
2019	1 421 174 (4.0) [3.8-4.2]	177 830 (3.6) [3.4-3.9]	

Abbreviation: SMD, standardized mean difference.

^a^ *P* < .001 for all comparisons.

^b^ Other race and ethnicity is derived from the race/ethnicity variable in the National Inpatient Sample (NIS) and includes Asian or Pacific Islander, Native American, and other defined by the NIS.

**Figure 1.**
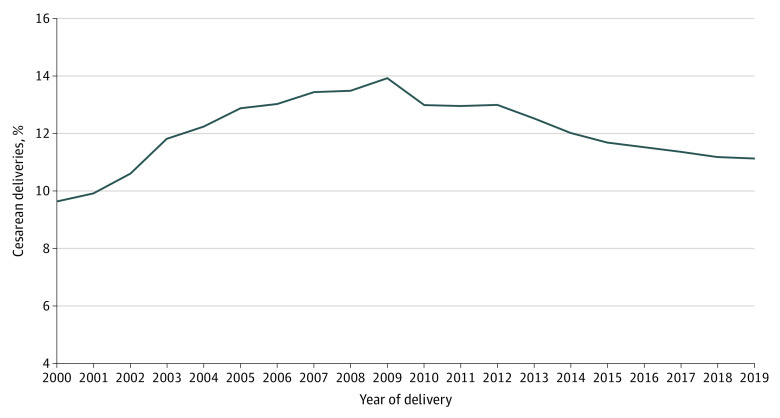
Trends in the Proportion of Cesarean Deliveries Among Low-risk Deliveries Graph shows the proportion of cesarean deliveries among low-risk deliveries in the National Inpatient Sample from 2000 to 2019.

Cesarean delivery rates increased from 9.7% to 13.9% between 2000 and 2009, and then decreased from 13.0% to 11.1% between 2012 and 2019 (Figure 1). The joinpoint analysis demonstrated 2 trend-change points. From 2000 to 2005, the AAPC for cesarean delivery was 6.4% (95% CI, 5.2% to 7.6%). The AAPC then plateaued between 2005 and 2009 (AAPC, 1.2%; 95% CI, −1.2% to 3.7%) before decreasing from 2009 to 2019 (AAPC, −2.2%; 95% CI, −2.7% to −1.8%) (eTable 2 in Supplement 1).

**Figure 2.**
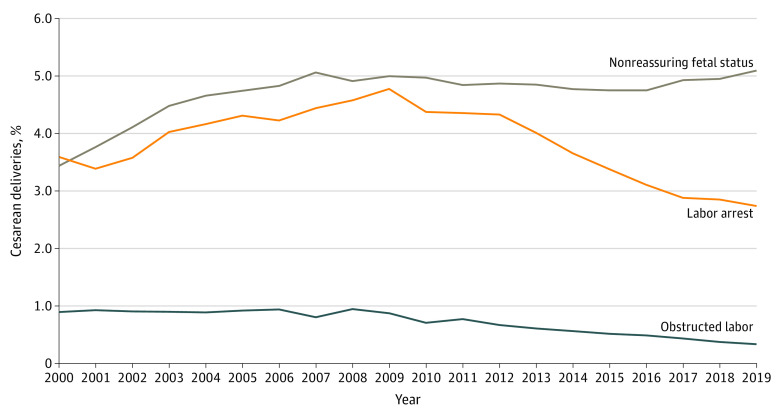
Trends in the Proportion of Cesarean Deliveries Among Low-risk Deliveries With Specific Diagnoses Graph shows the proportion of cesarean delivery hospitalizations with diagnoses of nonreassuring fetal status, labor arrest, and obstructed labor among low-risk deliveries in the National Inpatient Sample from 2000 to 2019.

**Figure 3.**
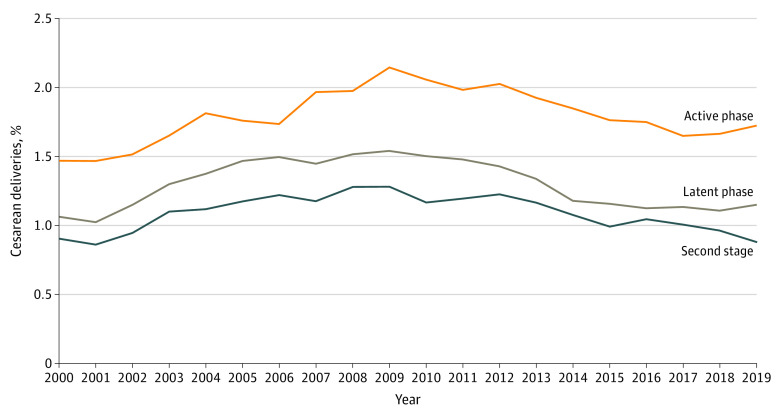
Trends in the Proportion of Cesarean Deliveries Among Low-risk Deliveries With Labor Arrest, by Stage of Labor Graph shows the proportion of cesarean delivery hospitalizations with second stage arrest, active phase arrest, and latent phase arrest among low-risk deliveries in the National Inpatient Sample from 2000 to 2019.

Evaluating specific indications, cesarean delivery with a diagnosis of nonreassuring fetal status increased from 3.4% of deliveries at low risk for cesarean birth in 2000 to 5.1% in 2019 (AAPC, 2.1%; 95% CI, 1.7% to 2.5%) (Figure 2 and eTable 2 in Supplement 1). Cesarean births with a diagnosis of labor arrest increased from 3.6% in 2000 to a peak of 4.8% in 2009 (AAPC 2000-2009, 3.8%; 95% CI, 2.7% to 4.8%), before decreasing to 2.7% in 2019 (AAPC 2009-2019, −5.6%; 95% CI, −6.6% to −4.6%). Cesarean births with obstructed labor decreased from 0.9% of deliveries in 2008 to 0.3% of deliveries in 2019, with joinpoint regression demonstrating a negative AAPC from 2009 onward (AAPC 2009-2019, −8.6%; 95% CI, −10.0% to −7.1%). Cesarean deliveries associated with latent phase, active phase, and second stage labor arrest diagnoses all decreased in the latter study period (Figure 3). During the first half of the study (2000-2009), cesarean deliveries for labor arrest increased for the active phase (from 1.5% to 2.1%), latent phase (from 1.1% to 1.5%), and second stage (from 0.9% to 1.3%) and then decreased from 2010 to 2019 (active phase, from 2.1% to 1.7%; latent phase, from 1.5% to 1.2%; and second stage, from 1.2% to 0.9%). The AAPC for latent phase arrest was −7.0% (95% CI, −12.1% to −1.7%) between 2011 and 2015. The AAPC for active phase arrest was −2.5% (95% CI, −3.3% to −1.6%) between 2009 and 2019. The AAPC for second stage arrest was −3.1% (95% CI −4.1% to −2.1%) between 2008 and 2019 (eTable 2 in Supplement 1).

In evaluating adjusted odds for cesarean delivery accounting for demographic and hospital characteristics, patients were at increased odds for cesarean delivery if they were aged 35 to 39 years compared with 25 to 29 years (aOR, 1.27; 95% 1.25-1.28), if they delivered in a hospital in the South compared with the Northeast (aOR, 1.11; 95% CI, 1.07-1.15), and if they were non-Hispanic Black compared with non-Hispanic White (aOR, 1.23; 95% CI, 1.20-1.25). Evaluating adjusted odds for year of delivery, odds for cesarean delivery increased from 2001 to 2009 before decreasing in the latter years of the study (Table 2).

**Table 2.** Unadjusted and Adjusted Logistic Regression Models for Cesarean Delivery

Variable	Unadjusted model, OR (95% CI)	Adjusted model, aOR (95% CI)^a^
Maternal race and ethnicity		
Hispanic	0.92 (0.89-0.96)	1.02 (0.99-1.05)
Non-Hispanic Black	1.19 (1.17-1.21)	1.23 (1.20-1.25)
Non-Hispanic White	1 [Reference]	1 [Reference]
Other^b^	1.04 (1.01-1.07)	1.08 (1.05-1.11)
Unknown	0.89 (0.86-0.92)	0.98 (0.96-1.01)
Age category, y		
15-17	1.11 (1.1-1.13)	1.17 (1.15-1.18)
18-24	1.00 (0.99-1.01)	1.03 (1.03-1.04)
25-29	1 [Reference]	1 [Reference]
30-34	1.07 (1.06-1.08)	1.05 (1.04-1.06)
35-39	1.30 (1.28-1.31)	1.27 (1.25-1.28)
Payer status		
Medicare	1.13 (1.09-1.18)	1.06 (1.02-1.10)
Medicaid	0.84 (0.82-0.85)	0.79 (0.78-0.80)
Private insurance	1 [Reference]	1 [Reference]
Self-pay	0.72 (0.68-0.75)	0.67 (0.64-0.70)
Other	0.88 (0.85-0.91)	0.85 (0.82-0.88)
Median income quartile by ZIP code		
1	1.02 (0.99-1.05)	1.00 (0.97-1.02)
2	0.96 (0.93-0.98)	0.98 (0.96-1.00)
3	0.98 (0.96-1.00)	1.00 (0.98-1.02)
4	1 [Reference]	1 [Reference]
Missing	1.05 (1.01-1.09)	1.06 (1.02-1.10)
Hospital location		
Rural	0.93 (0.90-0.96)	0.97 (0.94-1.00)
Urban		
Nonteaching	1 [Reference]	1 [Reference]
Teaching	0.94 (0.91-0.97)	0.92 (0.89-0.95)
Missing	0.91 (0.79-1.04)	0.88 (0.78-0.99)
Hospital region		
Northeast	1 [Reference]	1 [Reference]
Midwest	0.78 (0.75-0.81)	0.80 (0.77-0.82)
South	1.09 (1.05-1.13)	1.11 (1.07-1.15)
West	0.78 (0.75-0.82)	0.79 (0.76-0.82)
Year		
2000	1 [Reference]	1 [Reference]
2001	1.03 (0.98-1.08)	1.04 (1.00-1.09)
2002	1.11 (1.06-1.17)	1.12 (1.07-1.17)
2003	1.25 (1.18-1.33)	1.27 (1.21-1.34)
2004	1.31 (1.24-1.38)	1.33 (1.26-1.39)
2005	1.38 (1.30-1.47)	1.40 (1.33-1.48)
2006	1.40 (1.33-1.48)	1.45 (1.37-1.52)
2007	1.45 (1.38-1.54)	1.48 (1.41-1.56)
2008	1.46 (1.37-1.55)	1.50 (1.41-1.58)
2009	1.51 (1.42-1.61)	1.54 (1.45-1.63)
2010	1.40 (1.31-1.49)	1.43 (1.35-1.51)
2011	1.39 (1.31-1.48)	1.41 (1.34-1.49)
2012	1.40 (1.33-1.47)	1.41 (1.35-1.48)
2013	1.34 (1.28-1.41)	1.35 (1.30-1.42)
2014	1.28 (1.22-1.34)	1.31 (1.25-1.37)
2015	1.24 (1.18-1.30)	1.26 (1.21-1.32)
2016	1.22 (1.16-1.28)	1.24 (1.19-1.30)
2017	1.20 (1.14-1.26)	1.22 (1.17-1.28)
2018	1.18 (1.12-1.24)	1.20 (1.15-1.25)
2019	1.17 (1.12-1.23)	1.19 (1.14-1.25)

Abbreviations: aOR, adjusted odds ratio; OR, odds ratio.

^a^ The adjusted model included maternal age category, race, payer, income quartile, hospital location and teaching status, hospital region, and year of delivery.

^b^ Other race and ethnicity is derived from the race/ethnicity variable in the National Inpatient Sample (NIS) and includes Asian or Pacific Islander, Native American, and other defined by the NIS.

## Discussion

### Main Findings

This cross-sectional study found that among a national sample of patients at low risk for cesarean delivery, cesarean delivery rates increased from 2000 to 2009 and then decreased from 2010 to 2019. Cesarean deliveries associated with nonreassuring fetal status increased over the study period. In comparison, cesarean deliveries associated with labor arrest and obstructed labor increased during the first half of the study period from 2000 to 2009, but then decreased from 2010 to 2019. Decreases in cesarean delivery rates were noted separately for arrest during the latent phase of labor, the active phase of labor, and the second stage of labor.

### Clinical Implications

This analysis found that cesarean delivery rates decreased coincident with both the publication of findings from the Consortium on Safe Labor on longer physiologic labor length and new society guidance on specific criteria for diagnosing labor disorders.^1,3^ It is possible that the trends demonstrated in this study represent uptake of clinical management recommendations based on contemporary labor data by Zhang et al,^28^ which demonstrate slower normal labor progress than the historical standards provided by Friedman.^29^ Smaller clinical studies^4,6,7,8^ have demonstrated varied findings when longer labor definitions are adopted into clinical management, with some centers demonstrating decreased cesarean deliveries performed for labor arrest and dystocia. Given that trends in decreasing low-risk cesarean deliveries began in 2010, it is possible that physician recognition of increasing population-level labor duration was already beginning to change clinical practice when society guidance was released.

Cesarean delivery rates in this analysis were lower than those reported in low-risk populations from natality data, which are typically defined as nulliparous, singleton, full-term, and vertex births.^30,31^ This differential was not surprising given that our analysis excluded additional risk factors for cesarean birth and included delivery hospitalizations of patients with prior vaginal births. Similar to prior analyses evaluating births at low risk for cesarean delivery in the NIS, our analysis found a modest increase in the likelihood of cesarean delivery associated with demographic factors and hospital characteristics, including older maternal age, private insurance, and hospitals in the South.^32^

A key finding of this analysis was that although overall cesarean birth rates decreased, particularly in the setting of labor disorder diagnoses, cesarean births with a diagnosis of nonreassuring fetal status increased. Nomenclature and interpretation of intrapartum electronic fetal heart rate (FHR) monitoring also underwent changes over this study period. In 2008, a workshop including the Eunice Kennedy Shriver National Institute of Child Health and Human Development, the American College of Obstetricians and Gynecologists, and the SMFM led to recommendations to adopt a 3-tiered system for the categorization of FHR patterns.^33,34^ Most labors include category II FHR tracings, which are challenging from a management standpoint because of the uncertainty about the fetal status and, thus, remain the subject of ongoing research and quality improvement efforts.^35^ It is possible that current evidence and nomenclature related to intrapartum FHR interpretation may result in identification of a larger number of fetuses deemed at indeterminate risk for abnormal acid-base status. Further reduction of cesarean delivery rates in patients at low risk for cesarean delivery may require optimization of FHR interpretation. An additional factor potentially contributing to increasing diagnosis of nonreassuring fetal status may be longer labor occurring in the later study period. It is possible that some patients who would have undergone cesarean delivery on the basis of lower thresholds for diagnosing labor arrest subsequently developed nonreassuring fetal status diagnoses later in labor. Review of clinical data could be performed to determine the potential contribution of extended labor length to nonreassuring fetal status diagnoses.

### Strengths and Limitations

Strengths of this study include the use of a large, nationally representative population, allowing for meaningful statistical comparisons. This analysis spanned 20 years, allowing for assessment of trends over time and producing results generalizable on a population basis for the US. The ability to stratify labor arrest by stage of labor represents an additional study strength.

Our study also has several limitations. First, we relied on administrative hospital discharge data and not clinical records.^36^ Administrative data limitations for this study include that we were not able to control for factors such as parity and prior vaginal deliveries and that some diagnoses with separate codes such as labor arrest and obstructed labor may not be entirely distinct clinical entities. In addition, we were not able to perform medical record reviews to confirm that the diagnoses associated with delivery hospitalizations were, in fact, the clinical indications for cesarean delivery. The associations and trends in this analysis could be confirmed with large observational clinical studies or analyses of electronic health record data. Second, we excluded patients with chronic conditions associated with cesarean delivery that are increasing on a population basis from the low-risk study group.^14,15^ Although the prevalence of conditions such as chronic hypertension and preeclampsia are likely increasing in the obstetric population, more frequent diagnoses in the NIS may also be secondary to improved ascertainment and, thus, may be a source of unmeasured confounding. However, that differential trends were noted for cesarean births with varying diagnoses supports trends in differential clinical management. Third, the NIS went through 2 important changes during the study period, first in 2012 when the sampling approach changed, and second in 2015 when billing switched from *ICD-9-CM* to *ICD-10-CM *codes. It is possible that these changes could have affected some of the trends in our analysis. Fourth, absent longitudinal health records, we are not able to account for the complex interaction of health factors, maternal age, and use of assisted reproductive technology, such as in vitro fertilization. The use of in vitro fertilization, along with differentials in maternal age, may contribute to differential risk for placental dysfunction and cesarean delivery for fetal indications. These relationships could be disentangled with more granular data that better capture these factors.^37,38^ Fifth, we organized cesarean delivery diagnoses into a hierarchical, mutually exclusive, framework. In our analyses, we presumed that if a diagnosis for nonreassuring fetal status was present, that was the indication for cesarean delivery. In clinical practice, however, the decision for cesarean delivery may incorporate both abnormal labor progress and concerns regarding fetal status. Data sets with more granular clinical detail, including FHR monitoring parameters, labor progress measurements over time, and physician documentation, could be used to better model clinical decision-making. Sixth, this analysis is not able to analyze the effects of changes in clinical management on maternal and neonatal outcomes. Full evaluation of risks and benefits associated with changes in labor management would require more granular clinical data and linked neonatal information. Seventh, because the unit of analysis in the NIS is the individual delivery hospitalization, we are not able to account for multiple deliveries occurring to the same patient.

## Conclusions

In summary, this cross-sectional study found that low-risk cesarean deliveries are decreasing, that labor arrest diagnoses associated with cesarean delivery in this population are decreasing, and that nonreassuring fetal status diagnoses are increasing. These findings suggest countervailing trends, with changes in labor abnormality definitions resulting in decreased cesarean delivery and changes in intrapartum fetal tracing categorization resulting in increased cesarean delivery rates. Future reductions in cesarean deliveries among patients at low risk for cesarean delivery may be dependent on improved assessment of intrapartum fetal status.

## Notes

Supplement 1. **eTable 1.** Diagnosis Codes for Cesarean Indications **eTable 2.** Joinpoint Models With Average Annual Percent Change Estimates

Supplement 2.
Data Sharing Statement
