# Supplementary material for: Cesarean Delivery Trends Among Patients at Low Risk for Cesarean Delivery in the US, 2000-2019
Source: JAMA Netw Open. 2023 Mar 29;6(3):e235428. doi: 10.1001/jamanetworkopen.2023.5428 (PMC10061237; doi:10.1001/jamanetworkopen.2023.5428)
Supplement: Supplement 1. — eTable 1. Diagnosis Codes for Cesarean Indications eTable 2. Joinpoint Models With Average Annual Percent Change Estimates [file jamanetwopen-e235428-s001.pdf]

Supplemental Online Content

Frappaolo AM, Logue TC, Goffman D, et al. Cesarean delivery trends among patients at low risk for cesarean delivery in the US, 2000-2019. *JAMA Netw Open*. 2023;6(3):e235428. doi:10.1001/jamanetworkopen.2023.5428

eTable 1. Diagnosis Codes for Cesarean Indications
eTable 2. Joinpoint Models With Average Annual Percent Change Estimates

This supplemental material has been provided by the authors to give readers additional information about their work.

© 2023 Frappaolo AM et al. *JAMA Network Open.*

eTable 1. Diagnosis Codes for Cesarean Indications

Category	Diagnosis Codes	Diagnosis Descriptions
Non-reassuring fetal status	**ICD-9:**<br>656.3, 656.30, 656.31, 656.33, 659.7, 659.70, 659.71, 659.73<br><br>**ICD-10:**<br>O36.83, O36.833x, O36.839x, 068, O76, O77.9	• Fetal distress affecting management of mother<br>• 656.30 – Fetal distress, affecting management of mother, unspecified as to episode of care or not applicable<br>• 656.31 – Fetal distress, affecting management of mother, delivered, with or without mention of antepartum condition<br>• 656.33 – Fetal distress, affecting management of mother, antepartum condition or complication<br>• 659.7 – abnormality in fetal heart rate or rhythm<br>• 659.70 – abnormality in fetal heart rate or rhythm, unspecified as to episode of care or not applicable<br>• 659.71 – abnormality in fetal heart rate or rhythm, delivered, with or without antepartum condition<br>• 659.73 – abnormality in fetal heart rate or rhythm, antepartum condition or complication<br><br>• O36.83 – maternal care for abnormality of fetal heart rate or rhythm<br>• O36.833x – maternal care for abnormality of fetal heart rate or rhythm, third trimester<br>• O36.839x – maternal care for abnormality of fetal heart rate or rhythm, unspecified trimester<br>• O68 Labor and delivery complicated by abnormality of fetal acid-base balance<br>• O76 – abnormality in fetal heart rate or rhythm complicating labor and delivery<br>• O77.9 – Labor and delivery complicated by fetal stress, not otherwise specified
Labor arrest (non-specific)	**ICD-9:**<br>661.2, 661.20, 661.21, 661.23, 662, 662.0, 662.00, 662.01, 662.03, 662.1, 662.10, 662.11, 662.13	• 661.2 – other and unspecified uterine inertia<br>• 661.20 – other and unspecified uterine inertia, unspecified as to episode of care or not applicable<br>• 661.21 – other and unspecified uterine inertia, with or without antepartum condition<br>• 661.23 - other and unspecified uterine inertia, antepartum condition or complication

© 2023 Frappaolo AM et al. *JAMA Network Open.*

	**ICD-10:**<br>O62.2, O62.9, O63.0, O63.9	• 662 – long labor<br>• 662.0 – prolonged first stage of labor<br>• 662.00 – prolonged first stage of labor, unspecified as to episode of care or not applicable<br>• 662.01 – prolonged first stage of labor, delivered, with or without antepartum condition<br>• 662.03 – prolonged first stage of labor, antepartum condition or complication<br>• 662.1 – prolonged labor unspecified<br>• 662.10 – unspecified prolonged labor, unspecified as to episode of care or not applicable<br>• 662.11 – unspecified prolonged labor, delivered, with or without antepartum condition<br>• 662.13 – unspecified prolonged labor, antepartum condition or complication<br><br>• O62.2 – other uterine inertia<br>• O62.9 – abnormality of forces of labor, not otherwise specified<br>• O63.0 – prolonged first stage<br>• O63.9 – prolonged labor, not otherwise specified
Labor arrest (Latent phase arrest)	**ICD-9:**<br>661.0, 661.00, 661.01, 661.03<br><br>**ICD-10:**<br>O62.0	• 661.0 – primary uterine inertia<br>• 661.00 – primary uterine inertia, unspecified as to episode of care or not applicable<br>• 661.01 – primary uterine inertia, delivered, with or without mention of antepartum condition<br>• 661.03 – primary uterine inertia, antepartum condition or complication<br><br>• O62.0 – primary inadequate contractions
Labor arrest (Active phase arrest)	**ICD-9:**<br>661.1, 661.10, 661.11, 661.13<br><br>**ICD-10:**<br>O62.1	• 661.1 – active phase of labor arrest<br>• 661.10 – secondary uterine inertia, unspecified as to episode of care or not applicable<br>• 661.11 – secondary uterine inertia, delivered, with or without mention of antepartum condition<br>• 661.13 – secondary uterine inertia, antepartum condition or complication

© 2023 Frappaolo AM et al. *JAMA Network Open.*

		• O62.1 – arrested active phase/secondary uterine inertia
Labor arrest (Second stage arrest)	**ICD-9:**<br>652.5, 652.50, 652.51, 652.53, 662.2, 662.20, 662.21, 662.23<br><br>**ICD-10:**<br>O32.4, O32.4XX0, O63.1, O75.81	• 652.5 – high fetal head at term<br>• 652.50 – high fetal head at term, unspecified as to episode of care or not applicable<br>• 652.51 – high fetal head at term, with or without mention of antepartum condition<br>• 652.53 – high fetal head at term, antepartum condition or complication<br>• 662.2 – prolonged second stage of labor<br>• 662.20 – prolonged second stage of labor, unspecified as to episode of care or not applicable<br>• 662.21 – prolonged second stage of labor, with or without mention of antepartum condition<br>• 662.23 – prolonged second stage of labor, antepartum condition or complication<br><br>• O32.4 – high head at term/failed descent of head<br>• O32.4XX0 – head at term, not applicable or unspecified<br>• O63.1 – prolonged second stage<br>• O75.81 – maternal exhaustion
Labor arrest (Failed induction)	**ICD-9:**<br>659.0, 659.1, 659.00, 659.01, 659.03, 659.10, 659.11, 659.12<br><br>**ICD-10:**<br>O61.0, O61.1, O61.8, O61.9	• 659.0 – failed surgical induction of labor<br>• 659.1 – failed induction of labor<br>• 659.00 – failed mechanical induction of labor, unspecified as to episode of care or not applicable<br>• 659.01 – failed mechanical induction of labor, delivered, with or without antepartum condition<br>• 659.03 – failed mechanical induction of labor, antepartum condition or complication<br>• 659.10 – failed medical induction of labor<br>• 659.11 – failed medical or unspecified induction of labor, delivered, with or without antepartum condition<br>• 659.12 – failed medical or unspecified induction of labor, antepartum condition or complication<br><br>• O61.0 – failed medical induction of labor<br>• O61.1 – failed instrumental/mechanical induction of labor

© 2023 Frappaolo AM et al. *JAMA Network Open.*

		• O61.8 – other failed induction of labor<br>• O61.9 – failed induction of labor, unspecified
Obstructed labor/ Disproportion	**ICD-9:**<br>653, 653.0, 653.00, 653.01, 653.03, 653.1, 653.10, 653.11, 653.13, 653.2, 653.20, 653.21, 653.23, 653.3, 653.30, 653.31, 653.33, 653.4, 653.40, 653.41, 653.43, 653.5, 653.50, 653.51, 653.53, 653.6, 653.60, 653.61, 653.63, 653.7, 653.70, 653.71, 653.73, 653.8, 653.80, 653.81, 653.83, 653.9, 653.90, 653.91, 653.93, 660.10, 660.11, 660.13,<br>660.20, 660.21, 660.23<br><br>**ICD-10:**<br>O65.0, O65.1, O65.2, O65.3, O65.4, O65.5, O65.8, O65.9, O33, O33.1, O33.2, O33.3, O33.3XX0, O33.3XX1, O33.3XX9, O33.4, O33.4XX0, O33.4XX1, O33.4XX9, O33.5, O33.5XX0, O33.5XX1, O33.5XX9, O33.6, O33.6XX0, O33.6XX1,	• 653 Disproportion in pregnancy labor and delivery Non-specific code<br>• 653.0 Major abnormality of bony pelvis, not further specified, in pregnancy, labor, and delivery Specific code<br>• 653.00 Major abnormality of bony pelvis, not further specified, unspecified as to episode of care or not applicable<br>• 653.01 Major abnormality of bony pelvis, not further specified, delivered, with or without mention of antepartum condition<br>• 653.03 Major abnormality of bony pelvis, not further specified, antepartum condition or complication<br>• 653.1 Generally contracted pelvis in pregnancy labor and delivery<br>• 653.10 Generally contracted pelvis, unspecified as to episode of care or not applicable<br>• 653.11 Generally contracted pelvis, delivered, with or without mention of antepartum condition<br>• 653.13 Generally contracted pelvis, antepartum condition or complication<br>• 653.2 Inlet contraction of pelvis in pregnancy labor and delivery Specific code<br>• 653.20 Inlet contraction of pelvis, unspecified as to episode of care or not applicable<br>• 653.21 Inlet contraction of pelvis, delivered, with or without mention of antepartum condition<br>• 653.23 Inlet contraction of pelvis, antepartum condition or complication<br>• 653.3 Outlet contraction of pelvis in pregnancy labor and delivery<br>• 653.30 Outlet contraction of pelvis, unspecified as to episode of care or not applicable<br>• 653.31 Outlet contraction of pelvis, delivered, with or without mention of antepartum condition convert<br>• 653.33 Outlet contraction of pelvis, antepartum condition or complication

© 2023 Frappaolo AM et al. *JAMA Network Open.*

	O33.6XX9, O33.7, O33.7XX0, O33.7XX1, O33.7XX9, O33.8, O33.9	• 653.4 Fetopelvic disproportion Specific code 653.40 Fetopelvic disproportion, unspecified as to episode of care or not applicable<br>• 653.41 Fetopelvic disproportion, delivered, with or without mention of antepartum condition<br>• 653.43 Fetopelvic disproportion, antepartum condition or complication<br>• 653.5 Unusually large fetus causing disproportion<br>• 653.50 Unusually large fetus causing disproportion, unspecified as to episode of care or not applicable<br>• 653.51 Unusually large fetus causing disproportion, delivered, with or without mention of antepartum condition<br>• 653.53 Unusually large fetus causing disproportion, antepartum condition or complication<br>• 653.6 Hydrocephalic fetus causing disproportion<br>• 653.60 Hydrocephalic fetus causing disproportion, unspecified as to episode of care or not applicable<br>• 653.61 Hydrocephalic fetus causing disproportion, delivered, with or without mention of antepartum condition<br>• 653.63 Hydrocephalic fetus causing disproportion, antepartum condition or complication<br>• 653.7 Other fetal abnormality causing disproportion<br>• 653.70 Other fetal abnormality causing disproportion, unspecified as to episode of care or not applicable<br>• 653.71 Other fetal abnormality causing disproportion, delivered, with or without mention of antepartum condition<br>• 653.73 Other fetal abnormality causing disproportion, antepartum condition or complication<br>• 653.8 Disproportion of other origin in pregnancy labor and delivery<br>• 653.80 Disproportion of other origin, unspecified as to episode of care or not applicable

		• 653.81 Disproportion of other origin, delivered, with or without mention of antepartum condition<br>• 653.83 Disproportion of other origin, antepartum condition or complication<br>• 653.9 Unspecified disproportion in pregnancy labor and delivery<br>• 653.90 Unspecified disproportion, unspecified as to episode of care or not applicable<br>• 653.91 Unspecified disproportion, delivered, with or without mention of antepartum condition<br>• 653.93 Unspecified disproportion, antepartum condition or complication<br>• 660.10 Obstruction by bony pelvis during labor, unspecified as to episode of care or not applicable<br>• 660.11 Obstruction by bony pelvis during labor, delivered, with or without mention of antepartum condition<br>• 660.13 Obstruction by bony pelvis during labor, antepartum condition or complication<br>• 660.20 Obstruction by abnormal pelvic soft tissues during labor, unspecified as to episode of care or not applicable<br>• 660.21 Obstruction by abnormal pelvic soft tissues during labor, delivered, with or without mention of antepartum condition<br>• 660.23 Obstruction by abnormal pelvic soft tissues during labor, antepartum condition or complication<br>• O33 Maternal care for disproportion<br>• O33.1 Maternal care for disproportion due to generally contracted pelvis<br>• O33.2 Maternal care for disproportion due to inlet contraction of pelvis<br>• O33.3 Maternal care for disproportion due to outlet contraction of pelvis<br>• O33.3XX0 …… not applicable or unspecified O33.3XX1 …… fetus 1<br>• O33.3XX9 …… other fetus<br>• O33.4 Maternal care for disproportion of mixed maternal and fetal origin

© 2023 Frappaolo AM et al. *JAMA Network Open.*

		• O33.4XX0 …… not applicable or unspecified<br>• O33.4XX1 …… fetus 1<br>• O33.4XX9 …… other fetus<br>• O33.5 Maternal care for disproportion due to unusually large fetus<br>• O33.5XX0 …… not applicable or unspecified<br>• O33.5XX1 …… fetus 1<br>• O33.5XX9 …… other fetus<br>• O33.6 Maternal care for disproportion due to hydrocephalic fetus<br>• O33.6XX0 …… not applicable or unspecified<br>• O33.6XX1 …… fetus 1<br>• O33.6XX9 …… other fetus<br>• O33.7 Maternal care for disproportion due to other fetal deformities<br>• O33.7XX0 …… not applicable or unspecified<br>• O33.7XX1 …… fetus 1<br>• O33.7XX9 …… other fetus<br>• O33.8 Maternal care for disproportion of other origin<br>• O33.9 Maternal care for disproportion, unspecified<br>• O65.0 Obstructed labor due to deformed pelvis<br>• O65.1 Obstructed labor due to generally contracted pelvis<br>• O65.2 Obstructed labor due to pelvic inlet contraction<br>• O65.3 Obstructed labor due to pelvic outlet and mid-cavity contraction<br>• O65.4 Obstructed labor due to fetopelvic disproportion, unspecified<br>• O65.5 Obstructed labor due to abnormality of maternal pelvic organs<br>• O65.8 Obstructed labor due to other maternal pelvic abnormalities<br>• O65.9 Obstructed labor due to maternal pelvic abnormality, unspecified[1]

© 2023 Frappaolo AM et al. *JAMA Network Open.*

eTable 2. Joinpoint Models With Average Annual Percent Change Estimates

	Study interval	AAPC with 95% confidence intervals
**All cesarean deliveries**		
Overall	2000-2019	0.7% (0.1%, 1.3%)
Trend change points	2000-2005	6.4% (5.2%, 7.6%)
	2005-2009	1.2% (-1.2%, 3.7%)
	2009-2019	-2.2% (-2.7%, -1.8%)
**Trends in cesarean by indication**		
Nonreassuring fetal status		
Overall	2000-2019	2.1% (1.7%, 2.5%)
Trend change points	2000-2003	9.3% (7.8%, 10.8%)
	2003-2007	2.8% (1.5%, 4.1%)
	2007-2016	-0.6 (-0.9%, -0.3%)
	2016-2019	2.4% (0.9%, 3.9%)
Labor Arrest		
Overall	2000-2019	-1.3% (-1.9%, -0.6%)
Trend change points	2000-2009	3.8% (2.7%, 4.8%)
	2009-2019	-5.6% (-6.6%, -4.6%)
Obstructed Labor		
Overall	2000-2019	-4.8% (-5.7%, -3.9%)
Trend change points	2000-2009	-0.4% (-1.6%, 0.8%)
	2009-2019	-8.6% (-10.0%, -7.1%)
**Trends for in cesarean for labor arrest by stage of labor**		
Second stage arrest		
Overall	2000-2019	0.2% (-0.6%, 1.0%)
Trend change points	2000-2008	4.9% (3.4%, 6.5%)
	2008-2019	-3.1% (-4.1%, -2.1%)
Active phase		
Overall	2000-2019	0.7% (0.1%, 1.3%)
Trend change points	2000-2009	4.3% (3.4%, 5.3%)
	2009-2019	-2.5% (-3.3%, -1.6%)
Latent phase		
Overall	2000-2019	0.6% (-0.9%, 2.1%)
Trend change points	2000-2005	7.7% (5.3%, 10.2%)
	2005-2011	0.5% (-1.7%, 2.7%)
	2011-2015	-7.0% (-12.1%, -1.7%)
	2015-2019	0.0% (-3.9%, 4.1%)

© 2023 Frappaolo AM et al. *JAMA Network Open.*
