# Supplementary material for: Cesarean Delivery Trends Among Patients at Low Risk for Cesarean Delivery in the US, 2000-2019
Source: JAMA Netw Open. 2023 Mar 29;6(3):e235428. doi: 10.1001/jamanetworkopen.2023.5428 (PMC10061237; doi:10.1001/jamanetworkopen.2023.5428)
Supplement: Supplement 2. — Data Sharing Statement [file jamanetwopen-e235428-s002.pdf]

Data Sharing Statement

Frappaolo. Cesarean Delivery Trends Among Patients at Low Risk for Cesarean Delivery in the US, 2000-2019. *JAMA Netw Open*. Published March 29, 2023. doi:10.1001/jamanetworkopen.2023.5428

Data
Data available: No
